# Exerkines in Cardiovascular–Kidney–Metabolic Syndrome: An Integrative Framework for Mechanisms and Therapeutic Implications—A Narrative Review

**DOI:** 10.31083/RCM50762

**Published:** 2026-09-16

**Authors:** Hongyan Fan, Changlin Zhai, Chaojie He, Liang Shen, Haihua Pan, Wenwen Zheng

**Affiliations:** ^1^Department of Cardiology, The Affiliated Hospital of Jiaxing University, 314001 Jiaxing, Zhejiang, China; ^2^Department of Endocrinology, The Second Affiliated Hospital of Jiaxing University, 314001 Jiaxing, Zhejiang, China

**Keywords:** exerkines, cardiovascular–kidney–metabolic syndrome, myokines, hepatokines, adipokines, extracellular vesicles, exercise mimetics, cardiorenal syndrome, metabolic syndrome, precision medicine

## Abstract

Cardiovascular–kidney–metabolic (CKM) syndrome represents a pathophysiological continuum linking metabolic dysfunction, chronic kidney disease (CKD), and cardiovascular disease (CVD) through shared mechanisms, including insulin resistance, chronic inflammation, endothelial dysfunction, mitochondrial impairment, and maladaptive interorgan crosstalk. Although exerkines have emerged as key mediators of exercise-induced benefits, an integrated exerkine-centered framework spanning the CKM spectrum remains lacking. This review synthesizes current evidence on the multiorgan origins and biological roles of major exerkines, including myokines, adipokines, hepatokines, cardiokines, osteokines, lipid mediators, and extracellular vesicle–associated cargos. Moreover, extending beyond reductionist descriptions, we propose a higher-level mechanistic synthesis in which exerkines function as coordinated “signatures” across CKM phenotypes, converging on key regulatory hubs, particularly AMP-activated protein kinase (AMPK)–peroxisome proliferator-activated receptor gamma coactivator 1-alpha (PGC-1α)-mediated metabolic adaptation and the Klotho/fibroblast growth factor (FGF) axis that links renal, metabolic, and cardiovascular regulation. From a translational perspective, adiponectin, FGF21, apelin, and Klotho demonstrate the most consistent associations with cardiometabolic and renal outcomes in humans. However, clinical application remains constrained by uncertain causality, context-dependent resistance, and variability in assay performance. Overall, exerkines are best conceptualized as components of an integrated signaling network rather than isolated therapeutic targets. Meanwhile, future progress will depend on defining disease-stage-specific exerkine signatures and establishing associated causal relevance in humans.

## 1. Introduction

Cardiovascular–Kidney–Metabolic (CKM) syndrome represents a conceptual framework that integrates metabolic dysfunction, chronic kidney disease (CKD), and cardiovascular disease (CVD) into a unified pathophysiological network [1]. In CKM syndrome, traditional risk factors such as insulin resistance, obesity, and hypertension are tightly interwoven with renal impairment and cardiac dysfunction, driving a vicious cycle of organ crosstalk, inflammation, endothelial injury, and metabolic inflexibility [2,3,4]. This complex interplay exacerbates the progression of metabolic derangement, glomerular decline, and cardiovascular events [5].

Exercise has long been established as a cornerstone of prevention and treatment for cardiometabolic disorders [4,5]. Beyond improving fitness, reducing adiposity, and enhancing glucose control, its benefits extend to renal and cardiovascular protection. However, for many years, the molecular mediators that translate physical activity stimuli into systemic organ-protective effects have remained elusive. More recently, the discovery of exerkines—a broad class of exercise-induced signalling molecules—has provided new insights into how exercise confers multiorgan benefits [6]. Exerkines are bioactive factors released from multiple organs—including skeletal muscle, adipose tissue, liver, heart and other tissues—in response to acute or chronic exercise [7]. They exert their effects through autocrine, paracrine, and endocrine pathways, thereby regulating key processes implicated in CKM pathophysiology, such as energy metabolism, inflammation, mitochondrial biogenesis, endothelial function, and tissue remodelling. Importantly, exerkines are increasingly recognised not as isolated mediators, but as integral components of a dynamic interorgan communication network that coordinates systemic adaptations to physical activity [6,7,8].

Several recent reviews have summarised the biology of exerkines or their roles in individual cardiometabolic diseases. However, these studies have largely focused on single-organ perspectives or isolated disease domains. In contrast, the present review adopts the CKM framework to integrate cardiovascular, renal, and metabolic disorders into a unified disease continuum. Furthermore, it emphasises the concept of a multiorgan exerkine network, highlighting coordinated cross-talk between tissues rather than isolated signalling pathways. Importantly, this review also provides a translational perspective, discussing the potential and limitations of exerkines as biomarkers, mediators of exercise responsiveness, and emerging therapeutic targets. By addressing these gaps, we aim to provide a more integrative and clinically relevant understanding of exerkines in CKM syndrome.

Against this background, the present review aims to: (1) systematically summarise the major sources and biological characteristics of exerkines across multiple organs; (2) synthesise current evidence linking exerkines to the key components of CKM syndrome, including cardiovascular, renal, and metabolic dysfunction; and (3) critically evaluate their potential as biomarkers and therapeutic targets, with particular emphasis on their translational relevance and current limitations.

Fig. 1 illustrates the multiorgan exerkine network induced by exercise, highlighting the major organ sources and circulating mediators. Fig. 2 presents an integrated signalling model demonstrating how exerkines contribute to interconnected cardiovascular, renal, and metabolic processes within the CKM framework.

**Fig. 1.**
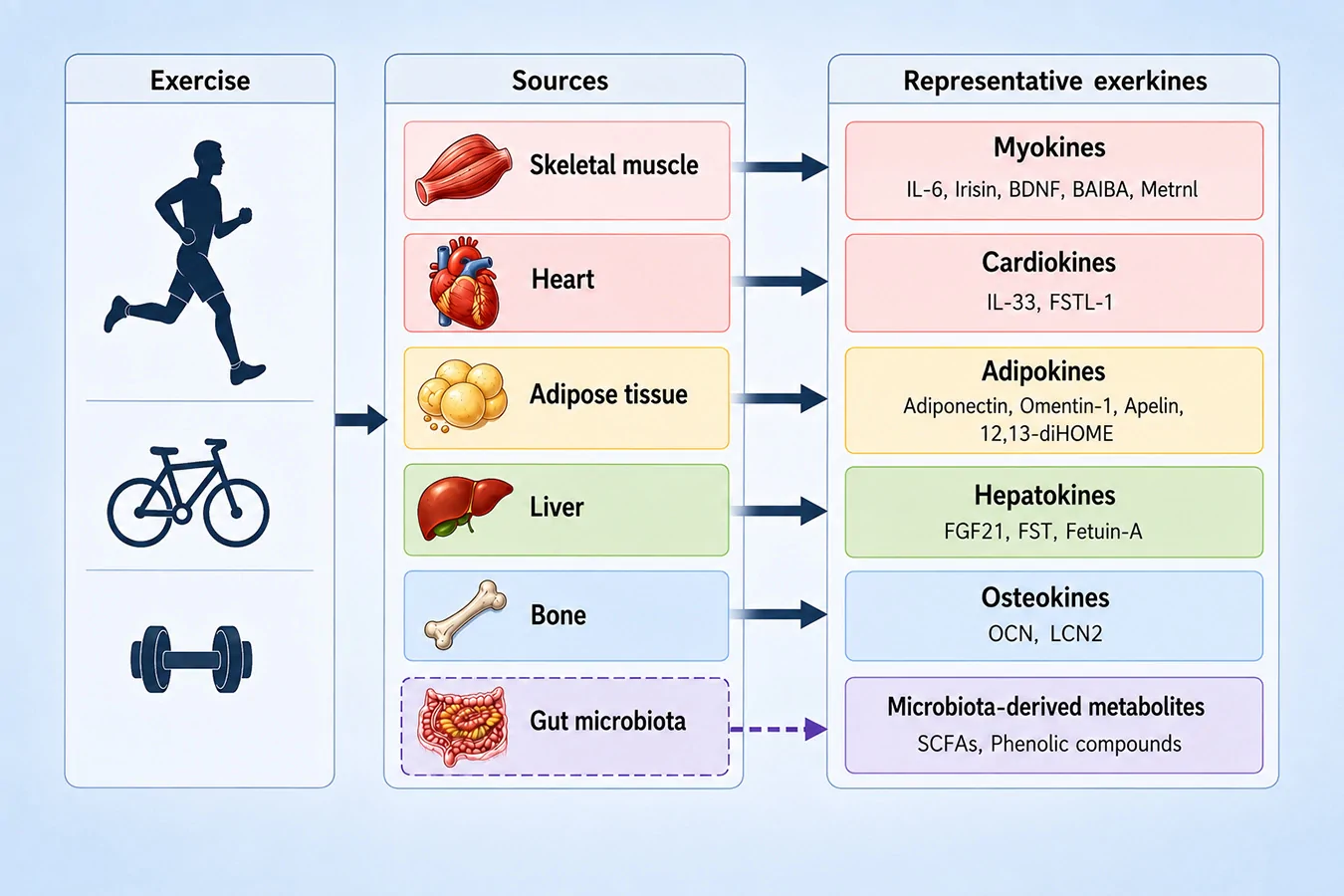
**Exercise-induced exerkine network across multiple organs**. Multiorgan sources and representative components of the exerkine network. Exercise stimulates endocrine and paracrine signalling from skeletal muscle, heart, adipose tissue, liver, and bone, resulting in the release of representative myokines, cardiokines, adipokines, hepatokines, and osteokines, respectively. Gut microbiota-derived metabolites are presented separately as an extended component of the exerkine network, with the dashed outline and arrow indicating their proposed and conceptually distinct role within this network. Whether microbiota-derived metabolites should be classified as exerkines remains debated. IL, interleukin; BDNF, brain-derived neurotrophic factor; BAIBA, β-aminoisobutyric acid; Metrnl, meteorin-like protein; FSTL-1, Follistatin-like protein 1; 12,13-diHOME, 12,13-dihydroxy-9-octadecenoic acid; FGF21, fibroblast growth factor 21; FST, follistatin; OCN, osteocalcin; LCN2, lipocalin-2; SCFAs, short-chain fatty acids.

**Fig. 2.**
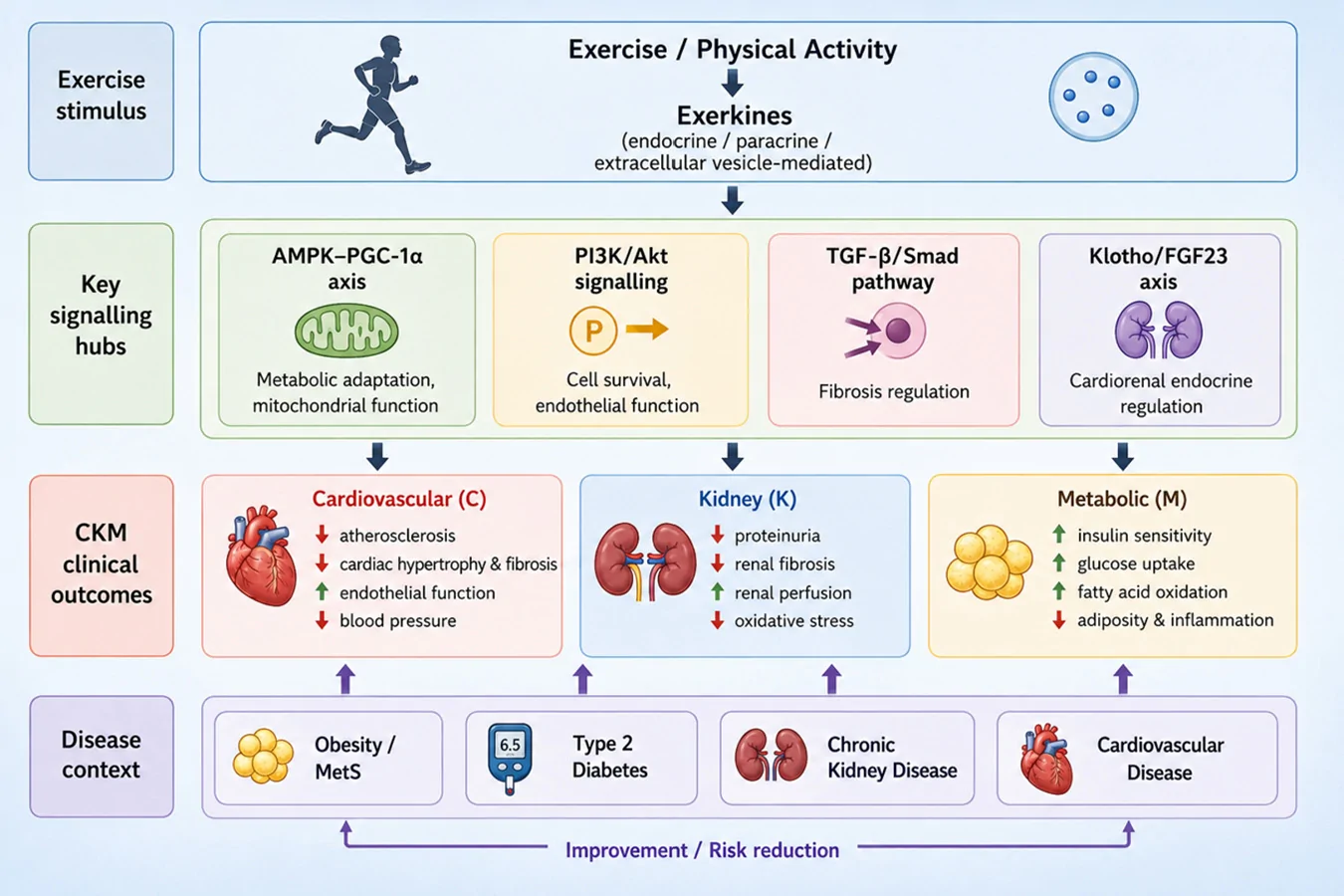
**Exerkine-mediated inter-organ signalling linking exercise to CKM pathophysiology**. Schematic overview of the integrative role of exercise-induced exerkines within the CKM continuum. Physical activity stimulates the release of exerkines, which act via key signalling hubs, including the AMPK–PGC-1α axis, PI3K/Akt pathway, TGF-β/Smad signalling, and the Klotho/FGF23 axis. These coordinated mechanisms regulate mitochondrial function, inflammation, endothelial integrity, and fibrotic responses. Collectively, these effects translate into clinically relevant improvements across CKM domains, including enhanced vascular function, reduced cardiac and renal fibrosis, improved insulin sensitivity, and optimised energy metabolism. Ultimately, these integrated adaptations contribute to risk reduction and disease modification in obesity, type 2 diabetes, chronic kidney disease, and cardiovascular disease. CKM, cardiovascular–kidney–metabolic; AMPK–PGC-1α, AMP-activated protein kinase–peroxisome proliferator-activated receptor gamma coactivator 1-alpha; PI3K, phosphatidylinositol 3-kinase; Akt, protein kinase B; TGF-β, transforming growth factor-β; Smad, small mother against decapentaplegic; FGF, fibroblast growth factor.

## 2. Survey Methodology

We performed a comprehensive literature search using the PubMed, Web of Science, and Scopus databases, covering studies published up to April 2026. The search strategy incorporated the following terms: “exerkines”, “myokines”, “adipokines”, “hepatokines”, “cardiokines”, “osteokines”, “gut microbiota–derived metabolites”, “cardiovascular disease”, “chronic kidney disease”, “metabolic syndrome”, and “CKM syndrome”. Both preclinical and clinical studies were included. Only articles published in English were considered, and additional relevant studies were identified through manual screening of review articles and the reference lists of eligible publications.

## 3. Sources of Exerkines: A Multiorgan Endocrine Network

Exerkines originate from various organs that undergo mechanical, metabolic, and endocrine adaptation during exercise. These tissues release cytokines, peptides, metabolites, and extracellular vesicle (EV)–packaged signals that mediate local and systemic responses. This section focuses on the organ-specific origins of exerkines and highlights those that have demonstrated or are increasingly recognised to have relevance to CKM-related outcomes, including cardiovascular function, renal dysfunction, and metabolic regulation.

### 3.1 Skeletal Muscle

Accounting for approximately 40–60% of human body weight, skeletal muscle serves as both the primary organ for locomotion and a significant endocrine organ. Contracting myofibres secrete numerous myokines that regulate metabolism, inflammation, mitochondrial function, and interorgan communication. Identified myokines include interleukin (IL)-6, IL-7, IL-15, irisin, myonectin, myostatin (MSTN), brain-derived neurotrophic factor (BDNF), meteorin-like protein (Metrnl), β-aminoisobutyric acid (BAIBA), and mitochondrial open reading frame of 12S rRNA-c (MOTS-c) [9].

Among the large number of identified myokines, several have been directly linked to CKM-related pathways. IL-6, irisin, BDNF, Metrnl, and BAIBA are among the best-characterised myokines. Acute exercise induces a marked increase in circulating IL-6, which in humans has been associated with enhanced glucose uptake and lipid metabolism [10]. However, chronically elevated IL-6 levels are linked to systemic inflammation and increased cardiometabolic risk [11]. Irisin and BAIBA have been shown, in both animal models and human studies, to improve insulin sensitivity, promote lipid oxidation, and enhance mitochondrial function, with emerging evidence suggesting additional roles in endothelial function and renal protection [12,13]. BDNF has been implicated in the maintenance of podocyte integrity and may be associated with the risk of chronic kidney disease in observational cohorts [14,15]. Metrnl has been implicated in the regulation of insulin sensitivity and inflammatory homeostasis and may contribute to metabolic improvement within the CKM framework [16,17,18]. In addition, skeletal muscle releases extracellular vesicles (EVs) enriched with microRNAs that regulate endothelial function, angiogenesis, and cardiac metabolism [19]. While preclinical evidence supporting these effects is substantial, human data remain limited and are largely confined to surrogate endpoints.

Overall, skeletal muscle-derived exerkines represent the most extensively studied group, with a relatively robust evidence base linking them to metabolic regulation, cardiovascular and renal outcomes within the CKM framework.

### 3.2 Heart

Cardiokines are proteins secreted by the heart into the circulation, where they play essential physiological roles in maintaining cardiac homeostasis or responding to myocardial injury, thereby influencing the development of cardiovascular disease. Several exercise-responsive cardiokines, including IL-33 and Follistatin-like protein 1 (FSTL-1), have been identified [20].

Preclinical studies suggest that IL-33 exerts anti-atherosclerotic and anti-inflammatory effects, including the modulation of macrophage function and the reduction of cardiomyocyte apoptosis [21,22,23,24,25,26]. Similarly, FSTL-1 has been shown to improve endothelial function, promote angiogenesis, and attenuate cardiac fibrosis in animal models [27,28,29,30,31]. However, evidence in humans remains limited, and the majority of data linking cardiokines to CKM-related outcomes are derived from experimental studies. Their role in integrated cardio–renal–metabolic regulation therefore remains to be fully elucidated.

### 3.3 Adipose Tissue

Adipose tissue is widely recognised as the largest endocrine organ in the human body and produces more than 600 bioactive molecules, which are collectively referred to as adipokines [32]. A growing body of evidence has demonstrated that these factors play pivotal roles in diverse biological and physiological processes, including appetite regulation, inflammation and immune responses, glucose and lipid metabolism, and blood pressure control. Although acute exercise has limited immediate effects on the secretion of adipokines, chronic exercise training can markedly alter their expression and release profiles. Several representative exercise-responsive adipokines include adiponectin, omentin-1, apelin, and 12,13-dihydroxy-9-octadecenoic acid (12,13-diHOME).

Adiponectin is supported by robust human evidence, with consistent associations with improved insulin sensitivity, reduced inflammation, and lower cardiovascular risk [33]. Apelin has been linked to vascular function, blood pressure regulation, and cardiac performance in both experimental and clinical settings [34]. 12,13-diHOME, a lipokine induced by exercise, enhances fatty acid uptake and mitochondrial function in skeletal muscle and has been associated with improved cardiometabolic profiles, although human evidence remains emerging [35]. Omentin-1 has been associated with insulin sensitivity and metabolic health; however, findings are heterogeneous across populations [36,37].

Overall, adipose-derived exerkines demonstrate relatively strong associations with the metabolic components of CKM syndrome, with more variable but progressively accumulating evidence for cardiovascular relevance.

### 3.4 Liver

The liver functions as both an energy-storage depot and an energy-utilising organ that becomes highly metabolically active during exercise to maintain whole-body energy homeostasis. Like myokines and adipokines, signalling molecules that are produced exclusively or predominantly by the liver and exert endocrine effects on extrahepatic tissues are referred to as hepatokines. Emerging in recent years as a novel group of exerkines, hepatokines are primarily proteins. These proteins are released from the liver during exercise or the postexercise recovery period, resulting in altered circulating levels, with some being upregulated while others are decreased. Currently identified exerkines secreted by the liver include fetuin-A, fibroblast growth factor 21 (FGF21), and follistatin (FST).

FGF21 is one of the most extensively studied hepatokines and has been linked, in both experimental and human studies, to lipid metabolism, insulin sensitivity, and cardiovascular risk [38,39,40,41]. However, circulating FGF21 levels are paradoxically elevated in obesity and cardiometabolic disease, which may reflect a state of FGF21 resistance rather than enhanced biological activity [42]. Fetuin-A has been associated with insulin resistance and vascular calcification in human studies, whereas FST has demonstrated metabolic regulatory effects primarily in experimental models [43,44,45,46].

Overall, hepatokines play a significant role in the metabolic and cardiovascular components of CKM syndrome, although their effects appear to be context-dependent and incompletely characterised in humans.

### 3.5 Bone

Bone-derived proteins, collectively termed osteokines, play a pivotal role in the regulation of skeletal metabolism and systemic homeostasis [47]; however, their association with CKM syndrome remains relatively underexplored. Osteocalcin (OCN) has been associated with glucose metabolism and insulin sensitivity in human studies, while lipocalin-2 (LCN2) has been shown in experimental models to influence appetite regulation and glucose homeostasis [48,49]. Compared with other exerkine sources, the evidence linking osteokines to CKM-related outcomes is less well established, and their role should therefore be considered emerging.

### 3.6 Gut Microbiota

Growing amounts of evidence have suggested that gut microbiota–derived metabolites represent a metabolically active extension of the exerkine network and contribute substantially to the systemic benefits elicited by physical activity. Exercise induces compositional and functional changes in the gut microbiome, leading to altered production of bioactive metabolites, including short-chain fatty acids (SCFAs) and phenolic compounds, which are reversed with training cessation [50]. These microbiota-derived metabolites have been associated with key CKM-related processes, including improved insulin sensitivity, modulation of systemic inflammation, and regulation of blood pressure in both animal models and human studies [51]. Furthermore, during exercise, specific gut microbes synthesise small‑molecule metabolites—such as 3‑hydroxyphenylacetic acid and 4‑hydroxybenzoic acid—that attenuate myocardial injury and promote functional recovery after myocardial infarction in mice [52]. This evidence suggests potential links to cardiovascular outcomes, although these findings remain preliminary.

Conceptually, whether microbiota-derived metabolites should be classified as exerkines remains debated, in this review, they are considered part of an extended exerkine network, representing a complementary axis through which exercise influences cardio–renal–metabolic homeostasis.

## 4. Exerkines in Relation to CKM Syndrome: From Interorgan Signalling to Disease Pathophysiology

Within the framework of CKM syndrome, exerkines constitute an inter‑organ endocrine network that connects skeletal muscle, adipose tissue, the liver, the cardiovascular system, and the kidneys. Through their regulation of energy metabolism, inflammation, mitochondrial function, endothelial homeostasis, and tissue remodelling, exerkines offer a mechanistic explanation for the systemic benefits conferred by physical activity. This section synthesises current evidence on the role of exerkines in the pathophysiology of CKM syndrome, thereby providing a foundation for a focused examination of their cardiovascular, renal, and metabolic actions (Table 1, Ref. [11,14,15,16,17,18,21,22,23,24,25,26,27,28,29,30,31,38,39,40,41,53,54,55,56,57,58,59,60,61,62,63,64,65,66,67,68,69,70,71,72,73,74,75,76,77,78,79,80,81,82,83,84,85,86,87,88,89,90,91,92,93,94,95,96,97]; Fig. 2).

**Table 1.** **The roles of exerkines in CKM syndrome**.

Name	Origin	Primary CKM domain	Signalling pathway	Main biological effects	Clinical relevance	Reference
IL-6	Myokine	C+M	AMPK, PI3K/Akt	Metabolic regulation; context-dependent inflammation	Human (context-dependent)	[11,53,54]
IL-33	Cardiokine	C	ST2L signalling, ERK1/2	Anti-atherosclerosis; anti-inflammatory	Preclinical	[21,22,23,24,25,26]
Irisin	Myokine	C+K+M	AMPK, PI3K/Akt, TGF-β/Smad	Endothelial protection; angiogenesis; anti-fibrosis	Human (inconsistent)	[55,56,57,58,59,60,79,80,81,89]
FSTL-1	Cardiokine	C	PI3K/Akt, AMPK, TGF-β/Smad	Endothelial protection; angiogenesis; anti-inflammation; anti-fibrosis	Preclinical	[27,28,29,30,31]
FGF21	Hepatokines	C+M	PI3K/Akt, TGF-β/Smad, ERK1/2	Lipid metabolism; cardiometabolic regulation; anti-fibrosis; stress-responsive (FGF21 resistance)	Human (paradox)	[38,39,40,41,61]
Apelin	Adipokines	C	AMPK, PI3K/Akt, APJ signalling	Vasodilation; angiogenesis; anti-apoptotic; antioxidative	Human (limited)	[62,63,64,65,66,67,68,69]
12,13-diHOME	Adipokines	C+M	NOS1–Ca^2+^ signalling, lipid uptake pathways	Fatty acid uptake; metabolic adaptation	Human (limited)	[70,97]
Klotho	Kidney	K	FGF23	Anti-inflammation; antioxidative; vascular protection	Human (consistent)	[71,72,73,74,75,76,77,78]
BAIBA	Myokine	K+M	AMPK, PPARα	Enhances mitochondrial function; anti-fibrosis; lipid oxidation; insulin sensitivity	Human (limited)	[82,83,85,86,87,88]
BDNF	Myokine	K	TrkB, AMPK	Stabilises podocyte cytoskeleton; anti-apoptotic	Human (limited)	[14,15,84]
Metrnl	Myokine	M	AMPK, PPARγ, PPARδ	Anti-inflammatory; adipose remodelling; insulin sensitivity	Preclinical	[16,17,18]
Adiponectin	Adipokines	M	AMPK, PPARα	Insulin sensitivity; anti-inflammatory; lipid oxidation	Human (consistent)	[90,91,92,93,94]
Omentin-1	Adipokines	M	AMPK	Insulin sensitivity; lipid oxidation	Human (limited)	[95,96]

CKM, cardiovascular–kidney–metabolic; IL, interleukin; AMPK, AMP-activated protein kinase; PI3K, phosphatidylinositol 3-kinase; Akt, protein kinase B; ERK, extracellular signal-regulated kinase; TGF-β, transforming growth factor-β; Smad, small mother against decapentaplegic; FSTL-1, Follistatin-like protein 1; FGF, fibroblast growth factor; 12,13-diHOME, 12,13-dihydroxy-9-octadecenoic acid; NOS, nitric oxide synthase; BAIBA, β-aminoisobutyric acid; PPAR, peroxisome proliferator-activated receptor; BDNF, brain-derived neurotrophic factor; TrkB, tropomyosin-related kinase B; Metrnl, meteorin-like protein; APJ, apelin receptor; FGF23, fibroblast growth factor 23; ST2L, transmembrane (long) isoform of suppression of tumorigenicity 2; C, cardiovascular; K, kidney; M, metabolic.

### 4.1 Exerkines in Cardiovascular Components of the CKM Spectrum

Within the CKM spectrum, cardiovascular disease represents a downstream organ component, characterised by endothelial dysfunction, vascular inflammation, mitochondrial impairment, maladaptive remodelling, and progressive loss of metabolic flexibility [6,98]. Increasing evidence suggests that exercise-induced exerkines help orchestrate coordinated cardio–renal–metabolic communication networks that influence these pathological processes [6,99]. Rather than acting as isolated signalling molecules, exerkines appear to function as components of a broader adaptive response that links skeletal muscle activity, adipose tissue remodelling, hepatic metabolism, and vascular homeostasis. Among the best-studied exerkines, several—including IL-6, IL-33, irisin, FSTL-1, FGF21, apelin, 12,13-diHOME, and a wide range of EV-associated miRNAs—exert direct effects on the cardiovascular system.

IL-6, recognised as the first myokine shown to respond to exercise, pioneered the conceptual framework for exerkines [100]. Circulating levels of IL-6 increase markedly during acute exercise and are released primarily from contracting skeletal muscle. Preclinical studies have demonstrated its direct role in cardioprotection, as exercise reduces myocardial infarct size and arrhythmias in a mouse model of ischaemia/reperfusion, and these effects are significantly attenuated in IL-6 knockout mice [53]. In further support of this cardioprotective role in humans, a randomised, double-blind, placebo-controlled trial in individuals with obesity demonstrated that pharmacologic blockade of IL-6 signalling via the receptor antagonist tocilizumab abolished the beneficial effects of aerobic exercise, including reductions in epicardial and pericardial adipose tissue and the expected increase in left ventricular mass [54]. However, the effects of IL-6 are highly context-dependent. In obesity, for example, chronic overproduction of IL-6 from adipose tissue exacerbates metabolic inflammation, promotes insulin resistance, and may ultimately increase cardiovascular risk [11]. These observations suggest that the biological significance of IL-6 depends strongly on the metabolic context and temporal pattern of exposure, highlighting the distinction between adaptive exercise signalling and chronic inflammatory activation within CKM syndrome.

Irisin represents one of the most extensively investigated exercise-responsive myokines in cardiovascular research. Experimental studies have linked irisin to improved endothelial function, reduced oxidative stress, and attenuation of adverse cardiac remodelling through pathways involving Akt and AMPK/endothelial nitric oxide synthase (eNOS) signalling [55,56,57,58,59,60]. Nevertheless, human observational studies have yielded inconsistent associations between irisin concentrations and cardiovascular risk, with some reports indicating inverse relationships with endothelial dysfunction and atherosclerosis, whereas others suggest no independent association after adjustment for adiposity and metabolic confounders [101,102,103]. In addition, assay variability and uncertainty regarding physiological versus pharmacological exposure continue to complicate interpretation of the available evidence [104]. Current evidence therefore supports a role for irisin within exercise-associated cardiovascular adaptation, although its independent clinical significance remains uncertain.

Several exerkines appear to influence cardiovascular biology through effects on endothelial function, vascular inflammation, and tissue remodelling. In experimental models, IL-33 has been linked to reduced vascular inflammation and attenuation of atherosclerotic progression, with additional evidence suggesting protective effects during myocardial stress and injury [21,22,23,24,25,26]. These actions are thought to involve the transmembrane (long) isoform of suppression of tumorigenicity 2 (ST2L)- and ERK1/2-related signalling pathways that regulate immune and cardiomyocyte responses under inflammatory conditions. FSTL-1 has likewise attracted attention because of its potential role in vascular repair and myocardial adaptation. Experimental studies indicate that FSTL-1 may promote endothelial recovery and angiogenesis, partly through PI3K/Akt/eNOS signalling, while also limiting fibrosis and ischaemia–reperfusion injury in the myocardium [27,28,29,30,31]. Exercise-induced increases in FSTL-1 expression have therefore been proposed as one mechanism linking physical activity to vascular and myocardial resilience. Nevertheless, evidence for both IL-33 and FSTL-1 remains predominantly preclinical, and their significance across different CKM stages in humans is still poorly defined.

FGF21 and apelin further illustrate the complexity of exercise-related signalling within the CKM continuum. Experimental studies have associated FGF21 with reduced oxidative stress, improved lipid handling, and suppression of vascular and myocardial injury pathways [38,39,40,41]. However, interpretation of circulating FGF21 levels in CKM syndrome remains challenging. Although exercise may transiently enhance FGF21 signalling, chronically elevated concentrations are frequently observed in obesity, diabetes, and cardiovascular disease, raising the possibility that increased circulating levels may reflect metabolic stress rather than sustained protective activity [61,105,106]. Similarly, apelin signalling has been implicated in angiogenesis, vascular remodelling, endothelial protection, and haemodynamic adaptation through coordinated regulation of AMPK/Akt-, PI3K/Akt-, and eNOS-related pathways [62,63,64,65,66,107]. Exercise training upregulates apelin signalling in both animal and human studies and has been associated with reduced arterial stiffness, improved vascular function, and favourable metabolic adaptation [66,67,68,69]. Even so, the biological effects of exerkines are unlikely to remain constant throughout CKM progression, and responses observed in experimental settings may not fully translate to advanced cardiometabolic disease in humans.

12,13-diHOME, a lipokine derived from brown adipose tissue (BAT), has emerged as an exercise-responsive mediator linking adipose tissue activity to cardiovascular and metabolic adaptation [70]. Experimental studies have associated 12,13-diHOME with improved cardiac mitochondrial function and haemodynamic adaptation under conditions of metabolic stress [35,70]. These effects may relate, at least in part, to modulation of NOS1-dependent calcium handling pathways. Circulating 12,13-diHOME levels are reduced in cardiovascular disease but increase markedly following acute exercise [108]. Exercise-induced increases in circulating 12,13-diHOME may therefore represent part of a broader adaptive response that coordinates substrate utilisation across adipose tissue, skeletal muscle, and the heart. Moreover, circulating levels of 12,13-diHOME have been associated with favourable metabolic profiles in some cohorts, but findings are not yet consistent across populations [109].

Exercise-induced EVs have emerged as important mediators of interorgan communication within the CKM network, particularly through the transfer of regulatory miRNAs involved in vascular and myocardial adaptation. Experimental studies suggest that EV-associated cargos contribute to angiogenesis, endothelial repair, mitochondrial adaptation, and cardiomyocyte survival through regulation of Akt/eNOS-, vascular endothelial growth factor (VEGF)-, and antiapoptotic signalling pathways [110,111,112,113,114]. These observations support the concept that EVs facilitate integrated adaptive communication among skeletal muscle, the vasculature, adipose tissue, and the heart during exercise rather than acting through isolated molecular pathways alone. Nevertheless, most evidence regarding EV-associated exerkines remains preclinical, and the functional significance of circulating EV profiles in human cardiovascular disease is incompletely understood [115]. In addition, substantial methodological heterogeneity in EV isolation, quantification, and characterisation continues to limit standardisation and translational interpretation across studies [115].

Taken together, current evidence suggests that exerkines contribute to cardiovascular adaptation within CKM syndrome primarily through integrated regulation of endothelial homeostasis, metabolic flexibility, mitochondrial function, inflammatory balance, and tissue remodelling. However, the translational significance of these findings remains incompletely established. Much of the mechanistic evidence is derived from experimental models, whereas human data remain heterogeneous and are frequently limited to surrogate biomarkers rather than hard cardiovascular outcomes. Accordingly, exerkines are perhaps best conceptualised not as isolated therapeutic targets, but as components of a dynamic adaptive signalling network linking exercise to systemic cardiovascular resilience across the CKM continuum.

### 4.2 Exerkines in Renal Components of the CKM Spectrum

Within the CKM continuum, CKD reflects progressive interactions among metabolic dysfunction, vascular injury, inflammation, and impaired tissue energetics. Hallmarks of CKD progression—including mitochondrial dysfunction, defective fatty acid oxidation, endothelial injury, and interstitial fibrosis—are increasingly recognised as potentially modifiable through physical activity. Emerging evidence suggests that exerkines participate in coordinated muscle–kidney–cardiovascular communication networks that influence these maladaptive processes, linking exercise to renal and systemic metabolic resilience.

Klotho is predominantly expressed in the kidney, particularly in distal tubular epithelial cells, with additional contributions from the parathyroid gland [116,117,118,119]. Exercise has been associated with increased circulating Klotho levels, potentially contributing to improved antioxidant defence, mineral metabolism, and endothelial function [71,72,73,74,75]. Mechanistically, Klotho acts as an essential coreceptor for fibroblast growth factor 23 (FGF23) signalling, thereby regulating phosphate handling and vitamin D metabolism while limiting vascular and renal injury associated with mineral dysregulation [73,76]. In addition, Klotho appears to attenuate oxidative stress, fibrosis, and metabolic dysfunction through modulation of cellular antioxidant and energy-regulating pathways [77,78,120]. Clinically, reduced circulating Klotho levels are consistently observed in CKD and are associated with adverse renal and cardiovascular outcomes [121,122]. These findings support a broader role for Klotho within the CKM continuum, although the clinical significance of exercise-induced changes in circulating Klotho remains uncertain. In particular, much of the available mechanistic evidence derives from recombinant or supraphysiological models rather than physiological exercise responses in humans [122].

Exercise-induced irisin has emerged as a potential mediator linking skeletal muscle activity to renal protection across CKM stages. Experimental studies indicate that irisin may attenuate albuminuria, podocyte injury, renal fibrosis, and ischaemia–reperfusion injury through coordinated regulation of TGF-β1- and AMPK-related pathways involved in metabolic stress and inflammatory responses [79,80,81,123,124]. In clinical settings, lower circulating irisin concentrations have been reported in patients with diabetic kidney disease and CKD [125,126,127,128], with additional associations observed between reduced irisin levels and adverse cardiometabolic profiles [129]. Nevertheless, current human evidence remains largely observational, and the translational relevance of exercise-induced irisin responses in CKD has yet to be established.

BAIBA, a small molecule released from skeletal muscle during exercise, has emerged as a potential link between muscle metabolism and renal function. Experimental studies indicate that BAIBA may improve mitochondrial function and metabolic capacity in podocytes, partly through pathways related to mitochondrial biogenesis and PGC-1α signalling [82]. Additional work suggests that BAIBA can attenuate oxidative stress and fibrotic responses in renal fibroblasts, including under conditions of angiotensin II (Ang II) stimulation [83]. Although these findings support a possible role for BAIBA in renal metabolic adaptation, current evidence remains almost entirely preclinical.

Although predominantly produced within the nervous system, BDNF is also expressed in the kidney, particularly in podocytes. Preclinical studies suggest that BDNF contributes to maintenance of glomerular structure and podocyte integrity through tropomyosin receptor kinase B signalling and downstream regulation of cytoskeletal pathways, including LIM domain kinase 1 (LIMK1)/cofilin-related mechanisms [14]. In addition, BDNF has demonstrated antiapoptotic effects in renal cells exposed to endoplasmic reticulum (ER) stress and toxic injury [15,84]. Clinical evidence indicates that reduced circulating BDNF levels are associated with an increased risk of incident CKD in individuals with preserved renal function at baseline, suggesting that systemic BDNF deficiency may predispose to renal decline [130]. These observations raise the possibility that reduced systemic BDNF activity may contribute to renal vulnerability during metabolic and inflammatory stress.

Collectively, these exerkines converge on several core mechanisms underlying CKD progression, including mitochondrial dysfunction, metabolic inflexibility, inflammation, fibrosis, endothelial injury, and podocyte damage. Importantly, many of these pathways are shared across cardiovascular, renal, and metabolic domains, reinforcing the concept of CKM syndrome as an interconnected multisystem continuum rather than a collection of isolated organ disorders. Current evidence increasingly supports the view that exercise-induced renal protection is mediated through coordinated interorgan signalling networks involving skeletal muscle, adipose tissue, the vasculature, and the kidney. Nevertheless, substantial translational challenges remain, particularly regarding context-dependent responses, disease-stage variability, and the relationship between physiological exercise-induced exerkine changes and the supraphysiological effects observed in experimental models.

### 4.3 Exerkines in Metabolic Dysfunction Within the CKM Spectrum

Within the CKM spectrum, metabolic syndrome (MetS) is considered an early metabolic phenotype rather than a separate disease entity. MetS is a systemic network disorder characterised by central obesity, insulin resistance, dyslipidaemia, low-grade inflammation, and mitochondrial dysfunction, all of which increase cardiovascular and renal risk. Exercise remains the most effective nonpharmacological intervention to modify this trajectory. Growing amounts of evidence have indicated that exerkines act as key endocrine mediators linking physical activity to improved metabolic homeostasis across multiple organs, including skeletal muscle, adipose tissue, liver, the heart, and the kidneys.

BAIBA, a small molecule released from skeletal muscle during exercise, has been implicated in the regulation of systemic metabolic homeostasis, particularly in relation to insulin resistance and dyslipidaemia. Its core biological actions, including AMPK activation and enhancement of mitochondrial metabolism, have been discussed in Section 3.2; here, we focus on its relevance to MetS. In preclinical models, BAIBA improves key features of MetS, including hepatic lipid accumulation, impaired fatty acid oxidation, and reduced insulin sensitivity in skeletal muscle and adipose tissue [85,86]. These effects are most evident under conditions of metabolic stress, such as lipid overload and endoplasmic reticulum stress, which are central to the development of MetS [87,88]. Human evidence remains limited and largely observational. Cross-sectional studies have reported inverse associations between circulating BAIBA levels and adiposity, insulin resistance, and adverse lipid profiles [131], although longitudinal and interventional data are still scarce [132]. Overall, current evidence suggests that BAIBA may contribute to exercise-associated improvements in metabolic flexibility and substrate utilisation.

Irisin has been closely linked to metabolic dysfunction across multiple CKM stages. Clinical studies have associated circulating irisin levels with central obesity, insulin resistance, dyslipidaemia, and other components of MetS [133]. However, reported relationships remain heterogeneous. Several cross-sectional studies have demonstrated inverse associations between irisin concentrations and body mass index, waist circumference, fasting glucose, and homeostatic model assessment of insulin resistance (HOMA-IR) [134], whereas elevated circulating levels have also been observed in individuals with established obesity or type 2 diabetes mellitus (T2DM) [89,135]. Rather than indicating uniformly beneficial signalling, these divergent findings may reflect altered endocrine responsiveness during chronic metabolic stress and progressive metabolic dysfunction.

Metrnl is an exercise-inducible secreted factor that has been implicated in the regulation of metabolic and inflammatory homeostasis, particularly in the context of MetS. Experimental studies suggest that Metrnl improves insulin sensitivity, attenuates adipose tissue inflammation, and promotes fatty acid utilisation through coordinated regulation of peroxisome proliferator-activated receptor (PPAR)γ-, AMPK-, and PPARδ-related pathways [16,17,18]. Human evidence for Metrnl in MetS remains limited and heterogeneous. Most available studies are cross-sectional, with some reporting lower circulating Metrnl levels in individuals with obesity or T2DM, whereas others have observed elevated or unchanged concentrations [136,137]. These discrepancies may reflect differences in adiposity, disease severity, and compensatory endocrine responses during metabolic dysfunction [138].

Adiponectin is a key adipose-derived hormone that contributes to systemic metabolic regulation and is responsive, albeit variably, to exercise interventions. Experimental studies have linked adiponectin to improved fatty acid oxidation, glucose metabolism, and insulin sensitivity through pathways involving AMPK and PPAR signalling [90,91,92,93,94]. In clinical studies, circulating adiponectin levels are consistently inversely associated with key components of MetS, including central obesity, insulin resistance, dyslipidaemia, and risk of T2DM [139,140]. Both cross-sectional and prospective cohort studies have demonstrated that lower adiponectin concentrations predict the development of MetS and T2DM, supporting its role as a robust biomarker of metabolic risk [141,142]. Although exercise interventions may increase adiponectin concentrations, responses appear highly influenced by baseline metabolic status, adiposity, and exercise characteristics [143,144].

Omentin-1 is an adipokine predominantly expressed in visceral adipose tissue and has been implicated in the regulation of insulin sensitivity and metabolic homeostasis. Although not considered a classical exercise-induced exerkine, its circulating levels appear to be influenced by metabolic status and lifestyle-related factors, including physical activity [95]. Experimental studies have indicated that omentin-1 exerts insulin-sensitising and metabolically favourable effects by increasing insulin-stimulated glucose uptake in adipocytes and activating AMPK signalling in skeletal muscle, thereby promoting energy expenditure and fatty acid oxidation [95,96]. In human studies, circulating omentin-1 levels are generally inversely associated with central obesity, insulin resistance, and adverse lipid profiles [145]. Lower concentrations are consistently observed in obesity and T2DM, although elevated levels have also been reported in conditions such as NAFLD, suggesting variable regulation across different metabolic states [95,146].

FGF21 is an endocrine hormone with broad metabolic regulatory functions, particularly in the context of glucose metabolism, lipid handling, and energy homeostasis. In addition to the mechanisms outlined above, FGF21 has been closely linked to metabolic dysfunction in MetS. Experimental studies suggest that FGF21 enhances glucose disposal, improves lipid metabolism, and attenuates insulin-resistant states partly through regulation of glucose transporter 1 (GLUT1)-mediated glucose uptake and adaptive metabolic signalling pathways. Clinically, circulating FGF21 levels are consistently elevated in obesity, MetS, T2DM, and NAFLD, supporting the concept that FGF21 may function as a stress-responsive biomarker in chronic metabolic disease rather than a uniformly protective endocrine signal [147,148,149,150].

12,13-diHOME is a lipid-derived signalling molecule increasingly recognised as a metabolically relevant lipokine linking adipose tissue activity to systemic energy homeostasis. Experimental studies have demonstrated that 12,13-diHOME enhances BAT function by promoting fatty acid uptake and lipoprotein lipase expression, leading to reduced circulating triglyceride levels and improved lipid tolerance in mice with diet-induced obesity [97]. These metabolic effects are closely associated with BAT activation and increased energy expenditure. In human studies, circulating 12,13-diHOME levels are inversely associated with body mass index, visceral adiposity, fasting insulin levels, HOMA-IR, and triglyceride concentrations, while showing positive associations with markers of lipolysis and fatty acid oxidation [97,108]. Nevertheless, reported associations with glucose metabolism remain less consistent across studies [35,151], and circulating levels may also be influenced by adiposity, physical activity, and BAT activity.

These findings suggest that exerkines operate within an interconnected signalling network through which exercise modulates insulin sensitivity, lipid metabolism, inflammation, and metabolic flexibility across CKM stages. Importantly, several exerkines display paradoxical or stage-dependent patterns, suggesting that chronic metabolic stress may induce compensatory endocrine activation or resistance states rather than sustained adaptive signalling. These observations support the concept that the metabolic benefits of exercise are unlikely to depend on isolated mediators alone, but instead arise from coordinated restoration of interorgan communication among skeletal muscle, adipose tissue, liver, the cardiovascular system, and the kidneys. Remaining translational challenges include substantial interindividual variability, disease-stage dependency, and uncertainty regarding the extent to which physiological exercise-induced exerkine responses reproduce the effects observed in experimental models.

### 4.4 Integrated Exerkine Network Across CKM Stages and Organ Systems

Although the above sections discuss cardiovascular, renal, and metabolic effects separately, CKM syndrome is fundamentally characterised by continuous and reciprocal interactions between organs rather than isolated dysfunction. Within this context, exerkines are better understood as components of a dynamic signalling network that links multiple tissues across the disease continuum [152].

Skeletal muscle appears to play a central initiating role in this network [153]. During exercise, muscle-derived factors influence hepatic glucose and lipid metabolism, modulate adipose tissue activity, and affect vascular function [153]. These primary signals are subsequently integrated at the level of the liver and adipose tissue, leading to secondary changes in hepatokines and adipokines, which further propagate metabolic information to the heart and kidney [153,154]. In this way, exerkine signalling is not unidirectional but involves iterative feedback loops between organs. Importantly, the biological role of exerkines is not static and seems to vary across the progression of CKM syndrome. In early metabolic disturbance, exercise-induced exerkines are generally associated with adaptive responses, including improved insulin sensitivity, enhanced mitochondrial function, and suppression of low-grade inflammation [155]. However, as disease advances, circulating levels of several exerkines become dissociated from their expected biological effects [61,135]. Under these conditions, exerkines may serve as markers of systemic stress as much as mediators of benefit.

Taken together, these observations suggest that the systemic effects of exercise are unlikely to be explained by individual exerkines in isolation. Instead, they reflect coordinated adjustments across an interorgan signalling network. This has important implications for translation: strategies that aim to mimic exercise by targeting single molecules may have limited efficacy, whereas approaches that restore network-level homeostasis may be more biologically plausible. A clearer understanding of how exerkine signalling evolves across CKM stages and how different organs integrate these signals will be essential for advancing this field.

## 5. Exercise Characteristics and Regulation of Exerkine Responses

While exerkines are increasingly recognised as important mediators of exercise-induced cardiometabolic adaptation, accumulating evidence suggests that their regulation is highly context-dependent and influenced not only by exercise prescription (such as exercise modality, intensity and duration) but also by the clinical characteristics of the individual [6]. In patients with CKM syndrome, factors such as disease stage, obesity phenotype, frailty, renal dysfunction, and exercise intolerance may substantially modify exerkine responses and thereby contribute to the marked heterogeneity observed in exercise outcomes.

Exercise modality seems to influence both the source and pattern of exerkine release. Aerobic exercise predominantly induces factors related to oxidative metabolism and substrate utilisation, including IL-6, BAIBA, and FGF21, reflecting increased energy demand and mitochondrial activation [54]. In contrast, resistance exercise is more closely associated with factors involved in muscle growth and remodelling, such as irisin, myostatin-related pathways, and certain extracellular vesicle–associated signals [156]. Nevertheless, these distinctions are not absolute, and combined aerobic–resistance programmes may induce broader and potentially complementary exerkine responses, particularly in patients with obesity, sarcopenia, or heart failure, in whom preservation of skeletal muscle mass and metabolic flexibility are both clinically relevant. Exercise intensity represents another important determinant. Acute high-intensity exercise typically induces a rapid and pronounced increase in circulating exerkines, particularly IL-6 and lactate-associated signalling molecules, which act as metabolic and inflammatory modulators [157]. By comparison, moderate-intensity exercise is generally associated with more sustained, albeit less pronounced, changes, which are thought to favour longer-term metabolic adaptation, particularly through cumulative mitochondrial and substrate utilisation responses [158]. Importantly, several exerkines appear to exhibit threshold-dependent responses, suggesting that a minimum exercise intensity or metabolic stimulus may be required to trigger biologically meaningful secretion. This issue may be particularly relevant in CKM populations with limited exercise capacity, where the ability to achieve sufficient physiological loading is often impaired. The temporal dimension of exercise further adds complexity to this relationship. Acute exercise induces transient elevations in many exerkines, whereas chronic training is associated with more stable adaptations, including altered baseline levels, improved sensitivity to signalling pathways, and changes in tissue responsiveness [156]. For example, repeated exercise exposure has been shown to attenuate excessive inflammatory responses while enhancing metabolic efficiency, suggesting a progressive shift from acute stress signalling towards adaptive remodelling [159]. However, the durability and clinical relevance of these adaptations remain incompletely understood, particularly in multimorbid CKM populations.

Exercise responsiveness across the CKM continuum appears to be highly stage-dependent. In early CKM stages, exercise predominantly exerts preventive and metabolic conditioning effects, whereas in advanced disease stages, the capacity to generate adaptive exerkine responses may become progressively impaired due to accumulated metabolic, inflammatory, and organ-specific dysfunction [160]. In Stage 0, exercise promotes insulin sensitivity, endothelial function, mitochondrial biogenesis, and metabolic flexibility, thereby helping to preserve optimal cardiometabolic health in individuals without overt metabolic abnormalities. During Stage 1, characterised primarily by excess adiposity, structured exercise interventions effectively reduce visceral fat accumulation, improve lipid handling, and enhance glucose regulation through coordinated myokine, adipokine, and hepatokine signalling. In Stage 2, encompassing established metabolic risk factors and early CKD, combined aerobic and resistance exercise may improve endothelial responsiveness, glycaemic control, vascular function, and renal haemodynamics, although the magnitude of these adaptations may already begin to vary substantially between individuals. By Stage 3, characterised by subclinical cardiovascular disease, targeted exercise interventions may help preserve vascular integrity, improve cardiac efficiency, and maintain metabolic resilience, particularly when initiated before irreversible structural dysfunction becomes established. Finally, in Stage 4 CKM syndrome, where overt cardiovascular disease, advanced CKD, frailty, or sarcopenia frequently coexist, exercise-based rehabilitation strategies such as moderate-intensity continuous training (MICT) and carefully supervised high-intensity interval training (HIIT) may improve functional capacity and quality of life, although physiological responsiveness is often attenuated compared with earlier disease stages.

Indeed, growing evidence suggests that exerkine responsiveness may differ substantially across clinical phenotypes and CKM stages. Patients with advanced obesity, heart failure, frailty, or CKD frequently exhibit mitochondrial dysfunction, chronic low-grade inflammation, endothelial impairment, anabolic resistance, and reduced metabolic flexibility, all of which may attenuate exercise-induced exerkine signalling and downstream tissue responsiveness [161,162,163]. In individuals with CKD, uraemic toxins, oxidative stress, muscle wasting, and reduced cardiorespiratory fitness may further attenuate exerkine production and downstream tissue responsiveness [161]. Similarly, frail or sarcopenic individuals may exhibit diminished skeletal muscle secretory capacity, potentially limiting the magnitude of beneficial myokine release during exercise [162]. These observations suggest that the biological response to exercise is unlikely to remain constant throughout CKM progression and may progressively decline with advancing metabolic and organ dysfunction.

Another important challenge relates to substantial interindividual variability in exercise responsiveness. Not all individuals exhibit comparable exerkine responses to a given exercise stimulus, even under standardised training conditions. Factors including age, sex, adiposity, baseline fitness, medication use, nutritional status, and gut microbiota composition may all contribute to heterogeneous exerkine profiles and variable physiological adaptation [164,165]. Moreover, significant translational uncertainties remain regarding the therapeutic relevance of exercise-induced exerkine alterations. Although many exerkines demonstrate beneficial effects in experimental models, it remains unclear whether the modest and transient physiological changes induced by exercise in humans are sufficient to reproduce these effects *in vivo* [6]. Furthermore, exerkines likely operate within an integrated signalling network rather than as isolated mediators, making causal relationships difficult to establish.

Taken together, current evidence suggests that exerkine responses are influenced not only by exercise characteristics but also by disease stage and individual biological context. Disease severity, frailty, obesity phenotype, renal dysfunction, and interindividual variability may all affect adaptive exerkine signalling, highlighting the need for phenotype-specific and stage-specific exercise strategies in CKM syndrome.

## 6. Exerkines as Therapeutic Targets in CKM Syndrome

### 6.1 Positioning Exerkines Within Current CKM Therapies

Current management of CKM syndrome is increasingly shaped by pharmacological agents with pleiotropic cardio–renal–metabolic benefits, including sodium–glucose cotransporter 2 inhibitors (SGLT2i), glucagon-like peptide-1 receptor agonists (GLP-1RA), and non-steroidal mineralocorticoid receptor antagonists [166]. These therapies have demonstrated robust clinical outcome benefits across cardiovascular and renal endpoints, thereby establishing a high benchmark for any emerging therapeutic strategies [167]. Within this context, exerkine-based approaches should be viewed cautiously. Rather than representing direct alternatives, they are more plausibly considered as complementary mechanisms that may converge on overlapping biological pathways, including metabolic regulation, anti-inflammatory signalling, and mitochondrial function. For instance, both exercise-induced exerkines and SGLT2i have been linked to improvements in substrate utilisation and systemic inflammation, although through distinct upstream triggers.

At present, there is insufficient clinical evidence to suggest that targeting individual exerkines could reproduce the broad and sustained benefits observed with established CKM therapies. Accordingly, it is more realistic to consider exerkine modulation as a potential adjunct to existing pharmacological and lifestyle interventions, rather than a replacement strategy.

### 6.2 Translational Potential, Current Limitations, and Safety Considerations

Growing evidence from preclinical and translational studies supports the concept that exercise-induced exerkines may represent a unifying biological axis across CKM syndrome. In this context, so-called “exercise mimetics”—pharmacological analogues designed to reproduce exerkine signalling—have been proposed as a potential strategy to emulate selected benefits of physical activity. These agents typically act as receptor agonists, thereby engaging downstream pathways involved in metabolic regulation, vascular function, and tissue adaptation [168].

Several candidates have shown encouraging results in early-stage studies. For instance, apelin administration has been reported to improve insulin sensitivity in individuals with obesity, while FGF21 analogues have progressed into clinical development for metabolic disorders, including obesity, T2DM, and nonalcoholic fatty liver disease [169,170,171]. In these settings, treatment has been associated with improvements in lipid profiles, reductions in hepatic fat content, and favourable changes in glucose and insulin homeostasis. Beyond metabolic effects, exerkines have also been implicated in cardiovascular adaptation. Apelin infusion has been shown to induce peripheral and coronary vasodilation and to increase cardiac output in both healthy individuals and patients with heart failure [172,173,174]. In addition, emerging evidence suggests that BDNF may influence fibrin clot properties in coronary artery disease [175]. Preclinical studies further indicate that irisin may exert renoprotective effects, including attenuation of glomerular injury and albuminuria through modulation of podocyte autophagy pathways [124].

However, these findings should be interpreted cautiously. Much of the current evidence remains derived from experimental models, whereas human studies are limited in scale, duration, and clinical endpoints. Even for exerkines that have entered early-phase clinical development, such as FGF21 analogues, available trials have focused predominantly on metabolic parameters, with limited evidence regarding hard cardiovascular or renal outcomes [176,177]. Moreover, circulating exerkine levels in established cardiometabolic disease may not necessarily reflect preserved biological responsiveness, further complicating translational interpretation [178]. Safety considerations also remain insufficiently characterised. Although early studies of FGF21 analogues have generally demonstrated acceptable tolerability, concerns have been raised regarding gastrointestinal adverse effects and possible impacts on bone metabolism [176]. Similarly, while apelin-based therapies have shown favourable haemodynamic effects, systematic long-term safety data in larger CKM populations are lacking [174]. More broadly, the long-term consequences of sustained pharmacological activation of exerkine pathways remain unknown. Given the pleiotropic roles of these molecules in metabolic, growth, and immune signalling, prolonged stimulation could theoretically result in unintended biological effects. Although such risks remain speculative, the absence of long-term outcome and safety data continues to represent a major translational limitation.

Exerkine-based therapies represent a biologically attractive concept within CKM syndrome, yet clinical translation remains preliminary. Current evidence is dominated by mechanistic and early-phase studies, while robust data on long-term efficacy, safety, and clinically relevant outcomes are still limited.

### 6.3 Biomarkers Versus Therapeutic Candidates

An important distinction should be made between exerkines that primarily serve as biomarkers and those that are being actively explored as therapeutic agents. Several circulating factors, including FGF21, FGF23, Klotho, and BDNF, appear to reflect underlying metabolic or organ stress and are increasingly being explored as biomarkers for risk stratification and disease monitoring, rather than as direct therapeutic targets [175,179]. In contrast, other exerkines, such as apelin and FGF21 analogues, have progressed into early-stage drug development, supported by preclinical efficacy and limited human data [169,170,171]. Additional candidates, including irisin and BAIBA, remain at a more exploratory stage, with ongoing debate regarding their physiological relevance and therapeutic feasibility [124,175].

This distinction is critical for avoiding overinterpretation of the current evidence. While biomarker studies provide important mechanistic insights, they do not necessarily imply therapeutic tractability. A clearer separation between these roles may help to align expectations and guide future research priorities.

## 7. Conclusion and Future Perspectives

Exerkines provide an integrative framework linking physical activity to coordinated cardio–renal–metabolic adaptation within the CKM continuum. Rather than acting in isolation, they function within a dynamic interorgan network regulating metabolism, inflammation, mitochondrial function, and tissue remodelling. Despite growing enthusiasm surrounding exerkine biology, current evidence remains heavily weighted towards mechanistic and surrogate endpoint studies. Whether modulation of exerkine signalling can translate into meaningful reductions in cardiovascular events, CKD progression, or mortality remains unknown. Several priorities should guide future research. First, omics-based profiling is needed to define exerkine signatures across CKM stages at a systems level. Secondly, causal inference approaches, including Mendelian randomisation, are required to distinguish mediators from biomarkers. Thirdly, modality-specific and dose–response exercise trials incorporating detailed exerkine profiling should clarify stimulus–response relationships. Finally, early-phase trials of exerkine mimetics in high-risk CKM populations are warranted, with emphasis on safety and clinically relevant outcomes.

This review has limitations. Assay variability across studies limits comparability, and publication bias towards positive mechanistic findings may overstate translational potential. In addition, as a narrative (non-systematic) review, the possibility of selection bias cannot be excluded. Overall, progress in this field will depend on moving from single-factor analyses towards integrated network-level understanding, supported by robust human studies.
